# Transcription Factors E2A, FOXO1 and FOXP1 Regulate Recombination Activating Gene Expression in Cancer Cells

**DOI:** 10.1371/journal.pone.0020475

**Published:** 2011-05-31

**Authors:** Zhengshan Chen, Yanna Xiao, Junjun Zhang, Jing Li, Yuxuan Liu, Yingying Zhao, Changchun Ma, Jin Luo, Yamei Qiu, Guowei Huang, Christine Korteweg, Jiang Gu

**Affiliations:** 1 Department of Pathology, Shantou University Medical College, Shantou, China; 2 Department of Pathology, Peking (Beijing) University Health Science Center, Beijing, China; Virginia Commonwealth University, United States of America

## Abstract

It has long been accepted that immunoglobulins (Igs) were produced by B lymphoid cells only. Recently Igs have been found to be expressed in various human cancer cells and promote tumor growth. Recombination activating gene 1 (RAG1) and RAG2, which are essential enzymes for initiating variable-diversity-joining segment recombination, have also been found to be expressed in cancer cells. However, the mechanism of RAG activation in these cancer cells has not been elucidated. Here, we investigated the regulatory mechanism of RAG expression in four human cancer cell lines by analyzing transcription factors that induce RAG activation in B cells. By RT-PCR, Western blot and immunofluorescence, we found that transcription factors E2A, FOXO1 and FOXP1 were expressed and localized to the nuclei of these cancer cells. Over-expression of E2A, FOXO1 or Foxp1 increased RAG expression, while RNA interference of E2A, FOXO1 or FOXP1 decreased RAG expression in the cancer cells. Chromatin immunoprecipitation experiments showed acetylation of RAG enhancer (Erag) and E2A, FOXO1 or FOXP1 were bound to Erag in vivo. These results indicate that in these cancer cells the transcription factors E2A, FOXO1 and FOXP1 regulate RAG expression, which initiates Ig gene rearrangement much in the way similar to B lymphocytes.

## Introduction

It has long been accepted that immunoglobulins (Igs) can only be expressed in mature B lymphocytes and plasma cells. However, recently several groups reported that Igs could also be produced by non-lymphoid lineage cells [1], including human cancer cells [2], [3], soft tissue tumor cells [4], neurons and glial cells of the central and peripheral nervous system [5], ocular epithelial and ganglion cells [6], mouse testicular spermatogenic cells and epididymal epithelial cells [7] and mouse lactating mammary gland epithelial cells [8]. Most of the research has thus far focused on Ig expression in cancer cells. The Recombination activating gene (RAG) has also been found expressed in cancer cells both at the mRNA and the protein levels and it is assumed to play a significant role in the synthesis of Igs by these cancer cells [2], [3], [9]. However, the regulatory mechanism of RAG expression in cancer cells has not yet been determined.

The variable regions of Ig genes are composed of one variable (V), one diversity (D), and one joining (J) gene segment, the arrangement of which results from V(D)J recombination [10]. RAG endonuclease is required for the initiation of the cleavage phase of V(D)J recombination [11]. RAG consists of two adjacent genes, RAG1 and RAG2, that synergistically induce V(D)J recombination [12]. Previous studies have shown that mice deficient in either RAG1 or RAG2 failed to initiate V(D)J rearrangement [13], [14]. RAG1 and RAG2 proteins together were found to be sufficient to cleave recombination substrates in cell free systems [15], [16]. In murine B cell development RAG expression occurs in two waves and is regulated by a network of transcription factors, including E2A, Ikaros, Pax5β, Foxo1, Foxp1, and NF-κB [17]. The first wave results in the rearrangement of the immunoglobulin heavy chain in pro-B cells. And the second wave of RAG expression leads to the assembly of immunoglobulin light chain in pre-B cells.

In addition to the RAG1 and RAG2 promoters, the RAG gene has also other regulatory elements, such as the proximal enhancer (Ep), the distal enhancer (Ed) and the RAG enhancer (Erag) [17], [18], [19], [20], [21], [22]. It is thought that the aforementioned transcription factors regulate RAG expression by binding to their corresponding regulatory sequences in B cells. Erag is the strongest enhancer regulating RAG expression. Targeted deletion of Erag in the mouse germline resulted in a 5-fold to 10-fold decrease in RAG expression and a partial block at the pro-B to pre-B transition [22]. E2A, Ikaros, Foxo1, Foxp1 and NF-κB were all shown to activate RAG expression by binding to Erag in murine B cells [22], [23], [24], [25], [26]. Pax5β was reported to activate RAG2 promoter in immature B cells [27]. Whether these transcription factors are also expressed in cancer cells and whether they have regulatory functions in the expression of RAG in such cells is worthy of investigation.

In this study, we first analyzed the protein and mRNA expressions of those transcription factors that have been found to be essential for RAG activation in B cells, including E2A (E47 and E12), FOXO1, FOXP1, Ikaros, NF-κB, and PAX5β, in four cancer cell lines. We then studied the localization of a number of these transcription factors (E2A, FOXP1, NF-κB and FOXO1) by immunofluorescence (IF). We found that E2A, FOXO1 and FOXP1 were expressed in cancer cells and localized to the nuclei of these cells. Over-expression of these three transcription factors significantly increased RAG expression. Functional inactivation of the genes of any of these three transcription factors by RNA interference decreased RAG expression. In vivo chromatin immunoprecipitation (ChIP) assay showed that the histone H3 of Erag was acetylated and that E2A, FOXO1, FOXP1 were bound to Erag in these cancer cells. These results indicate that transcription factors E2A, FOXO1 and FOXP1 activate the expression of RAG, which is critical for V(D)J recombination, in cancer.

## Materials and Methods

### Ethics statement

We didn't use any human or animal tissues in our study. So we didn't feel that ethics approval was necessary.

### Cell culture

The human lung cancer cell line A549, prostate cancer cell line PC3, breast cancer cell lines MCF-7, MDA-MB-231 and Burkitt lymphoma cell line Raji were obtained from the American Type Culture Collection (ATCC). A549, PC3, MCF-7 and MDA-MB-231 cells were cultured in Dulbecco's Modified Eagle's Medium (DMEM) with 10% FBS (Hyclone/Thermo Fisher Scientific Inc., Waltham, MA). Raji cells were cultured in RPMI 1640 (Invitrogen, Carlsbad, CA) with 10% FBS at 37°C in a humidified atmosphere with 5% CO2.

### RNA extraction and RT-PCR

Total RNA was extracted from the tumor cells using Trizol reagent (Invitrogen, Carlsbad, CA) and treated with RNase Free DNase (Invitrogen) to remove genomic DNA. Reverse transcription of the RNA was performed using the Superscript™ III First Strand Synthesis System (Invitrogen, Carlsbad, CA) following the manufacturer’s instructions. For the negative control, the reverse transcriptase was not added to the reaction mixture. Conventional or nested PCR was performed and the primers used in this study were listed in Table 1. For Ikaros, which has several different isoforms, nested PCR was used to increase the sensitivity and to better characterize the specific Ikaros isoforms [28]. The two isoforms of E2A, E47 and E12 were both amplified by PCR.

**Table 1:** Oligonucleotides used in this study.

Gene name	RT-PCR primers	Primer sequence 5’-3’	*product size (bp)*
Ikaros	External sense primer	CACATAACCTGAGGACCATG	255-945
	External antisense primer	AGGGCTTTAGCTCATGTGGA	
	Internal sense primer	ATGGATGCTGATGAGGGTCAAGAC	
	Internal antisense primer	GATGGCTTGGTCCATCACGTGG	
PAX5β	Sense primer	CCCGATGGAAATACACTGTAAGCAC	203
	Antisense primer	TTTTGCTGACACAACCATGGCTGAC	
P65	Sense primer	TCAATGGCTACACAGGACCA	308
	Antisense primer	CACTGTCACCTGGAAGCAGA	
E47	Sense primer	AGCAGTACGGACGAGGTGCTGTCCCTGG	162
	Antisense primer	CGCTTTGTCCGACTTGAGGTGCAT	
E12	Sense primer	ACCAGCCCAGACGAGGACGAGGACGACC	173
	Antisense primer	GGGCTTCTCGCTGTTGAGGTGCAG	
FOXO1	Sense primer	GCAGATCTACGAGTGGATGGTC	325
	Antisense primer	AAACTGTGATCCAGGGCTGTC	
FOXP1	Sense primer	TCAGTGGTAACCCTTCCCTTA	255
	Antisense primer	GTACAGGATGCACGGCTTG	
18S	Sense primer	AAACGGCTACCACATCCAAG	155
	Antisense primer	CCTCCAATGGATCCTCGTTA	
	RNAi sequence		
E2A		GGCGCAGUUCGGAGGUUCATT	
FOXP1		GCAGCAAGUUAGUGGAUUATT	
FOXO1		GCCCUGGCUCUCACAGCAATT	
	ChIP PCR primers		
Erag1	Sense primer	GCACTGCAAATGGCCTGTGAAC	197
	Antisense primer	TAGAGACCAGAGGGCTTAACATT	
Erag3	Sense primer	AAGCCTCTCTTTGCACCCTCAT	201
	Antisense primer	TTGAGTTGTCATTTCAGCCAAA	

### SDS-PAGE and Western blot

Cell lysates were prepared using RIPA buffer. About 40 µg of total cellular protein was separated on 5% to 10% SDS-PAGE gel. After electrophoresis, the separated proteins were transferred to a polyvinylidene difluoride membrane. The primary antibodies used included RAG1 (K-20), RAG2 (D-20), GAPDH (0411), E2A (Yae), FOXP1 (D35D10), NF-κB p65 (F-6), and FOXO1 (H-128). FOXP1 antibody was obtained from Cell Signaling Technology and the other antibodies were purchased from Santa Cruz Biotechnology. After incubation with the secondary antibodies (goat anti-mouse IgG-HRP or goat anti-rabbit IgG-HRP, Santa Cruz), the immunoblots were developed using Super ECL Plus Detection Reagent (Applygen Technologies, Beijing) and exposed to X-ray film according to the manufacturer’s protocol.

### Immunofluorescence

Cells were grown on slides in 6 well plates and fixed in 4% paraformaldehyde for 15 min at room temperature. Then the slides were incubated with 0.5% Triton X-100 for 10 min, and blocked for 1 hour in PBS containing 4% bovine serum albumin (BSA). The primary antibodies included E2A (Yae), FOXP1 (D35D10), NF-κB p65 (F-6) and FOXO1 (H-128). The detailed information for these antibodies were shown in table 2. Isotype controls were performed using normal mouse or rabbit IgG at the same concentration as the primary antibodies. After incubation at 4°C overnight and washing, the slides were incubated with the secondary antibody goat anti-mouse IgG-FITC (green signal) or goat anti-rabbit IgG-TRITC (red signal) at room temperature for 1 hour. After a final wash, slides were mounted with mounting media with DAPI (Vector Laboratories, Burlingame, CA) and examined under a fluorescence microscope (Carl Zeiss).

**Table 2:** Antibodies for immunofluorescence.

Primary antibody	Type	Concentration or dilution	Manufacturer
E2A (Yae)	Mouse, monoclonal	4 µg/ml	Santa Cruz Biotechnology, CA, USA
FOXO1 (H-128)	Rabbit, polyclonal	4 µg/ml	Santa Cruz Biotechnology, CA, USA
FOXP1 (D35D10)	Rabbit, monoclonal	1∶50	Cell Signaling Technology, MA, USA
NF-κB p65 (F-6)	Mouse, monoclonal	4 µg/ml	Santa Cruz Biotechnology, CA, USA

### Plasmids construction and transfection

The human E47 and E12 fragments were cloned into a pIRES2-EGFP plasmid by restriction enzyme Bgl II and EcoR I from plasmids MigR1-hE47 and MigR1-hE12, respectively, which were kind gifts from Dr. Barbara Kee of the University of Chicago. The pCDNAI/NEO-5’HA-Foxp1A plasmid which encoded murine Foxp1A protein was generously provided by Dr. Philip Tucker of the University of Texas at Austin [29]. The FOXP1 protein was very conserved between human and murine species (more than 90% identities), so we used this plasmid for our study. The pcDNA3-GFP-FOXO1;AAA plasmid was obtained from Addgene and it was prepared in Dr. William Sellers’ laboratory as described previously [30]. This plasmid contained a phosphosite mutation of FOXO1, which as a result hereof was no longer phosphorylated by Akt and could still localize to the nucleus and activate transcription in transfected cells [31]. Transient transfection assays of A549 and MCF-7 cancer cells were done using Fugene HD Transfection Reagent (Roche) according to the manufacturer’s instructions.

### RNA interference

Small interfering RNA (siRNA) directed against FOXO1 [32], FOXP1, E2A and nonspecific control siRNA (GenePharma, Shanghai, China) were transfected into A549 and MCF-7 cancer cells using Lipofectamine 2000 (Invitrogen) according to the manufacturer’s instructions. The siRNA sequences were listed in Table 1.

### ChIP

Chromatin crosslinking and immunoprecipitation were performed as described previously [23]. The anti-acetyl-histone H3 (06-599, Upstate Biotechnology), E2A (Yae), E47 (N-649), FOXP1 (D35D10) or FOXO1 (H-128) was used. Both the E2A (Yae) and E47 (N-649) antibody were used in ChIP for E2A. Normal rabbit IgG (sc-2027, Santa Cruz) or normal mouse IgG (sc-2025, Santa Cruz) was used as a negative control. Immunoprecipitated DNA sequences were analyzed with PCR and the primers were listed in Table 1.

## Results

### E2A, FOXO1, FOXP1 and NF-κB were expressed in cancer cell lines

**Figure 1:**
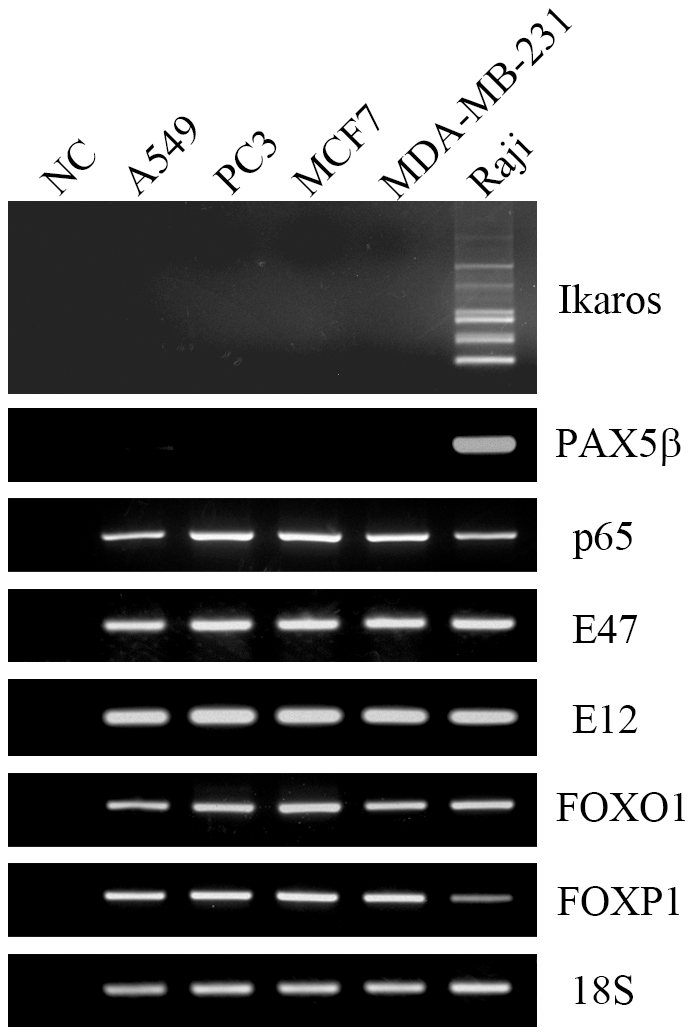
Gene transcript expression of Ikaros, PAX5β, NF-κB, E2A, FOXO1 and FOXP1 in cancer cell lines. 18S was used as an internal control. Raji was used as a positive control. DNase treated RNA without adding reverse transcriptase was used as a negative control.

**Figure 2:**
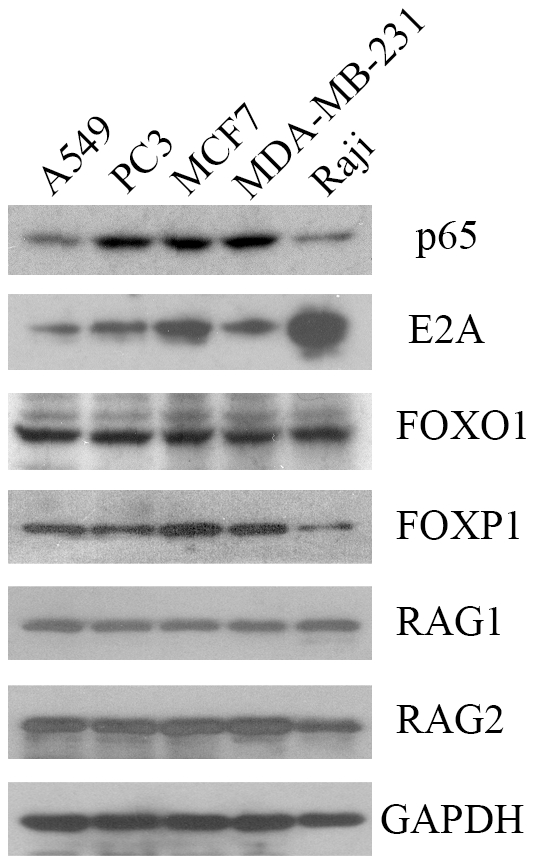
Western blot showing NF-κB p65, E2A, FOXO1, FOXP1, RAG expression in epithelial cancer cells. Raji cell was used as a positive control. GAPDH was used as an internal control.

RT-PCR results showed that FOXO1, FOXP1, NF-κB subunit p65 and both of the two isoforms of E2A (E47 and E12), were expressed in the cancer cells A549, PC3, MCF-7 and MDA-MB-231 (Figure 1). However, neither Ikaros nor PAX5β was detected in these cancer cell lines, whereas they could both be amplified from Raji cells. At the protein level, E2A, FOXO1, FOXP1 and NF-κB subunit p65 were all detected in the cancer cells with Western blot assay (Figure 2). Based on the molecular weight, the full length FOXP1 was found to be the main isoform of FOXP1 expressed in the cancer cells. We also confirmed the expression of RAG1 and RAG2 in these cancer cell lines (Figure 2).

### E2A, FOXO1 and FOXP1 were localized to the nuclei of cancer cells

**Figure 3:**
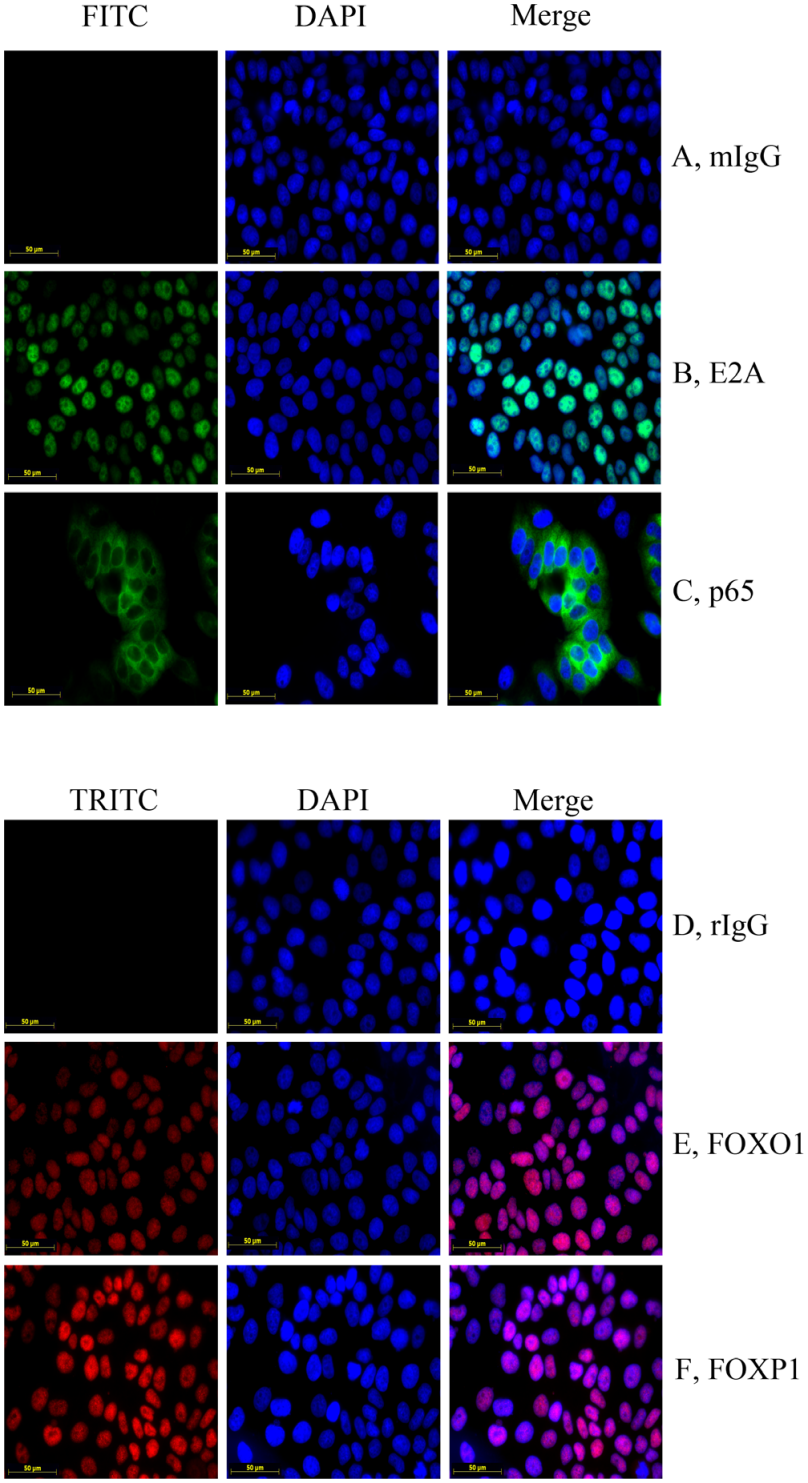
Immunofluorescence showing NF-κB p65, E2A, FOXO1 and FOXP1 localization in the MCF-7 cell line. A, normal mouse IgG was used instead of the primary antibody. B, the primary antibody was mouse anti-E2A. C, the primary antibody was mouse anti-NF-κB p65. A to C, the secondary antibody was goat anti-mouse IgG-FITC. D, normal rabbit IgG was used instead of the primary antibody. E, the primary antibody was rabbit anti-FOXO1. F, the primary antibody was rabbit anti-FOXP1. D to F, the secondary antibody was goat anti-rabbit IgG-TRITC. Similar results were obtained for the A549, PC3 and MDA-MB-231 cell lines (data not shown).

To study the localization of E2A, FOXO1, FOXP1 and NF-κB in cancer cells, IF was performed using the corresponding antibodies on the four cancer cell lines. The results showed that E2A and FOXP1 were predominantly localized to the nucleus, whereas NF-κB was exclusively localized to the cytoplasm of the cancer cells (Figure 3). FOXO1 was found to translocate between the nucleus and cytoplasm, with the location depending on the culture and growth conditions. When the cells were confluent, FOXO1 was mainly located in the nucleus, while it was mainly present in the cytoplasm when the cells were sparse. Since transcription factors need to be localized in the nucleus in order to regulate gene expression, we just focused on E2A, FOXO1 and FOXP1 in the second part of our study.

### Over-expression of E2A, FOXO1 or Foxp1 up-regulated RAG expression

**Figure 4:**
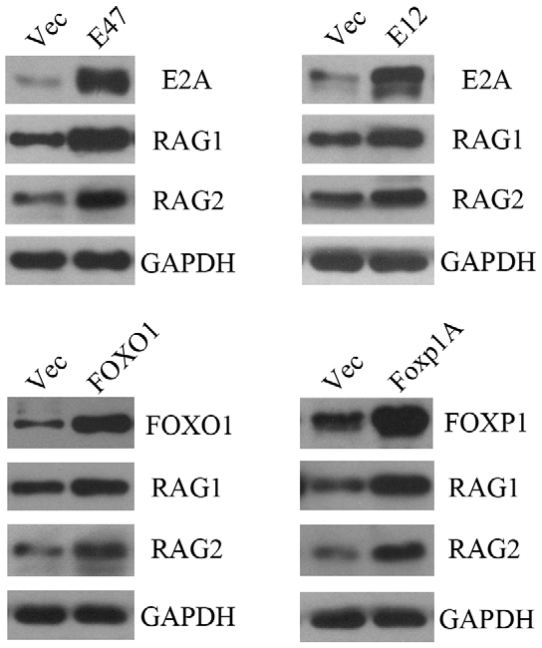
Transient over-expression of E47, E12, FOXO1 or Foxp1A increased RAG expression in MCF-7 cells. MCF-7 cells were transfected with the empty vector or transcription factor expression vector pIRES2-EGFP-hE47, pIRES2-EGFP-hE12, pcDNA3-GFP-FOXO1;AAA or pCDNAI/NEO-5’HA-Foxp1A. 48 hours later total protein from MCF-7 cells was collected and analyzed by Western blot. GAPDH was shown as a loading control. Experiments were repeated three times with similar results. Similar results were obtained for the A549 cell line (data not shown).

To explore the effect of transcription factors E2A, FOXO1 and FOXP1 on RAG expression, A549 and MCF-7 cells were transfected with the expression vector for E47, E12, Foxp1A or FOXO1. The empty vector was used as a negative control. Forty-eight hours after transfection, total protein was extracted for analysis of E2A, FOXP1, FOXO1 and RAG expression by Western blot assay. The results showed that transfection with the expression vector for E47, E12, Foxp1A or FOXO1 increased both RAG1 and RAG2 expressions (Figure 4). This data indicate that over-expression of E2A, FOXO1 or Foxp1 up-regulated the expression of RAG1 and RAG2 in MCF-7 cells. Similar results were obtained using the A549 cell line (data not shown).

### RNA interference of E2A, FOXO1 or FOXP1 down-regulated RAG expression

**Figure 5:**
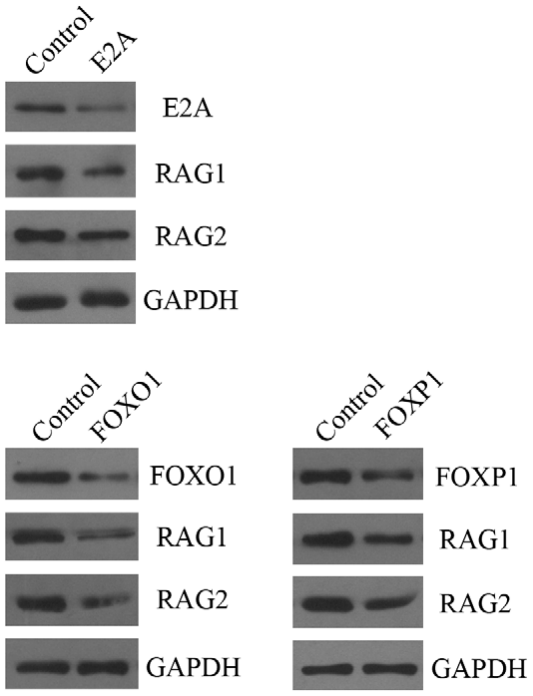
RNA interference of E2A, FOXO1 or FOXP1 decreased RAG expression in MCF-7 cells. MCF-7 cells were transfected with nonspecific control siRNA or siRNA sequence for E2A, FOXO1 or FOXP1. 48 hours later total protein from MCF-7 cells was collected and analyzed by Western blot. GAPDH was shown as a loading control. Experiments were repeated three times with similar results. Similar results were obtained for the A549 cell line (data not shown).

To further study the regulatory function of E2A, FOXO1 and FOXP1 on the expression of RAG, the siRNA sequences for E2A, FOXO1 or FOXP1 were transfected into A549 and MCF-7 cells. The nonspecific siRNA was used as the negative control. 48 hours after transfection, total protein was extracted for analyzing E2A, FOXP1, FOXO1 and RAG expression by Western blot assay. Transfection with siRNA sequence for E2A, FOXP1 or FOXO1 was found to decrease the expressions of both RAG1 and RAG2 (Figure 5), suggesting that silencing E2A, FOXO1 or FOXP1 genes down-regulates RAG1 and RAG2 expressions in MCF-7 cells. Similar results were obtained when using the A549 cell line (data not shown).

### E2A, FOXO1 and FOXP1 bound to Erag in vivo

**Figure 6:**
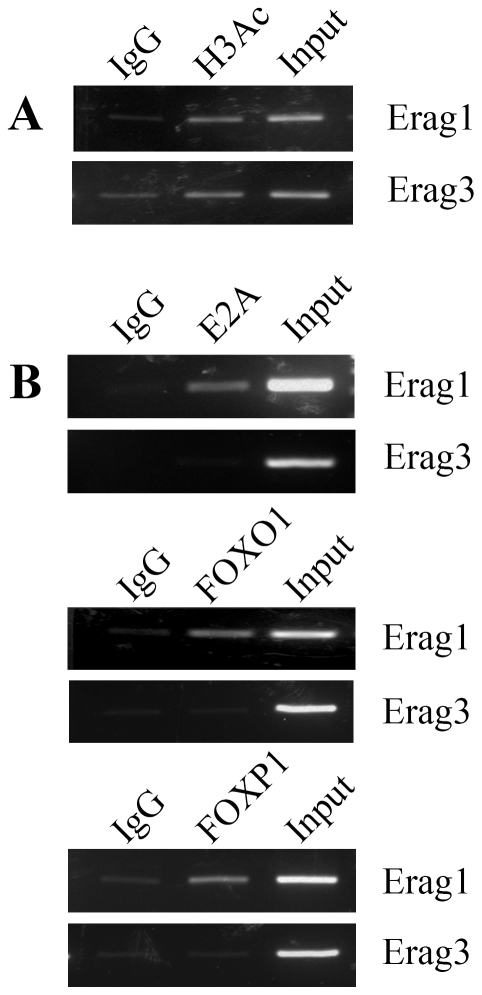
ChIP results showed histone H3 acetylation and E2A, FOXO1, FOXP1 binding of Erag in MCF-7. A, cross-linked chromatin isolated from MCF-7 cells was immunoprecipitated either with isotype control IgG or anti-acetyl-histone H3 antibody. The associated chromosomal DNA fragments were amplified with primers for Erag region 1 and 3. B, the antibody for the immunoprecipitation was anti-E2A, FOXO1 or FOXP1. Experiments were repeated three times with similar results. Similar results were obtained for A549 cell line (data not shown).

In murine B cells transcription factors E2A, Foxo1 and Foxp1 regulate RAG expression through binding to Erag, the enhancer regulating RAG gene expression. In order to investigate whether these transcription factors behave similarly in cancer cells, ChIP was performed on A549 and MCF-7 cells. The Erag region 1 contains three binding sites for E2A and one binding site for FOXO1 or FOXP1, whereas the Erag region 3 contains no binding site for E2A and one binding site for FOXO1 or FOXP1 [25]. ChIP results showed that histone H3 of both Erag region 1 and 3 was acetylated, indicating that Erag was in an open or activated state. In addition, transcription factors E2A, FOXO1 and FOXP1 were demonstrated to be bound to Erag region 1 but not to region 3 (Figure 6). For both cell lines similar results were obtained.

## Discussion

Recently several research groups reported that V(D)J recombination and RAG expression occurred in cancer cells. However, the mechanism controlling these phenomena in epithelial cells is currently unknown. In this study, in analogy with the molecular mechanism of RAG expression in B cells, we explored the regulatory mechanism of RAG expression in cancer cells by analyzing the transcription factors E2A, Ikaros, PAX5β, FOXO1, FOXP1 and NF-κB. Similar to their role in the activation of RAG expression in B cells, we found that E2A, FOXO1 and FOXP1 regulated RAG expression in cancer cells by binding to Erag.

E2A belongs to the class I helix-loop-helix (HLH) proteins, also known as E proteins because of their capacity to bind with relative high affinity to the palindromic DNA sequence CANNTG, referred to as an E box site [33], [34]. The E2A gene encodes for two E proteins, E12 and E47, which arise through alternative splicing of the exon encoding for the HLH domain [35]. E12 and E47 are primary transcription activators that function, in part, by recruiting the co-activator protein p300/CBP, which in turn recruits histone acetyltransferases and RNA polymerase II to the promoter or enhancers of target genes [36], [37]. E2A is believed to be a key regulator of B cell differentiation by activating the expression of RAG and other B lymphoid genes [34]. Over-expression of E47 has previously been found to activate RAG1 expression and IgH germ-line transcription in fibroblasts [38]. Ectopic expression of E2A, together with RAG1 and RAG2 promoted both IgH and IgL gene rearrangements in a non-lymphoid embryonic kidney cell line [39], [40]. Our finding that both RAG and E2A proteins were expressed in cancer cells contributes to the understanding of the mechanism of V(D)J recombination in neoplastic cells.

FOXO1 and FOXP1 belong to the family of Forkhead box proteins, which contain a common DNA-binding domain (DBD) termed the forkhead box or winged helix domain [41]. FOXO1 can translocate from the nucleus to the cytoplasm after being phosphorylated by Akt. Murine Foxp1 has four alternatively spliced isoforms, Foxp1A–Foxp1D [29]. Deregulation of FOXO1 and FOXP1 had been shown in many cancer types [41], [42]. Recently two studies showed that Foxo1 and Foxp1 regulated RAG expression in murine B cells [24], [25]. Here we have shown that FOXO1 and FOXP1 also have regulatory function in RAG expression in cancer cells.

In this study, we focused on a selected number of transcription factors and found that E2A, FOXO1 and FOXP1 regulated RAG expression in cancer cells. Whether there are additional transcription factors involved in RAG expression remains to be explored. In addition, whether there are more similarities in gene expression between B cells and cancer cells could be found using high throughput techniques. Previously, it was reported that Ig expression promoted tumor growth and progression [2], [43], [44]. In view of our results on regulatory factors activating RAG expression, Ig expression in cancer cells might be controlled by targeting the upstream transcription factors to eventually prevent tumor progression.
